# Treatments for brain metastases from EGFR/ALK-negative/unselected NSCLC: A network meta-analysis

**DOI:** 10.1515/med-2022-0574

**Published:** 2023-02-14

**Authors:** Chengkai Zhang, Wenjianlong Zhou, Dainan Zhang, Shunchang Ma, Xi Wang, Wang Jia, Xiudong Guan, Ke Qian

**Affiliations:** Department of Neurosurgery, Beijing Tiantan Hospital, Capital Medical University, Beijing 100071, China; Department of Neurosurgery, Beijing Neurosurgical Institute, Beijing 100071, China; Department of Neurosurgery, Beijing Tiantan Hospital, Capital Medical University, No. 119 West Road, Beijing 100071, China

**Keywords:** Bayesian network meta-analysis, non-small cell lung cancer, brain metastasis, immunotherapy, radiotherapy

## Abstract

More clinical evidence is needed regarding the relative priority of treatments for brain metastases (BMs) from EGFR/ALK-negative/unselected non-small cell lung cancer (NSCLC). PubMed, EMBASE, Web of Science, Cochrane Library, and ClinicalTrials.gov databases were searched. Overall survival (OS), central nervous system progression-free survival (CNS-PFS), and objective response rate (ORR) were selected for Bayesian network meta-analyses. We included 25 eligible randomized control trials (RCTs) involving 3,054 patients, investigating nine kinds of treatments for newly diagnosed BMs and seven kinds of treatments for previously treated BMs. For newly diagnosed BMs, adding chemotherapy, EGFR-TKIs, and other innovative systemic agents (temozolomide, nitroglycerin, endostar, enzastaurin, and veliparib) to radiotherapy did not significantly prolong OS than radiotherapy alone; whereas radiotherapy + nitroglycerin showed significantly better CNS-PFS and ORR. Surgery could significantly prolong OS (hazard ratios [HR]: 0.52, 95% credible intervals: 0.41–0.67) and CNS-PFS (HR: 0.32, 95% confidence interval: 0.18–0.59) compared with radiotherapy alone. For previously treated BMs, pembrolizumab + chemotherapy, nivolumab + ipilimumab, and cemiplimab significantly prolonged OS than chemotherapy alone. Pembrolizumab + chemotherapy also showed better CNS-PFS and ORR than chemotherapy. In summary, immune checkpoint inhibitor (ICI)-based therapies, especially ICI-combined therapies, showed promising efficacies for previously treated BMs from EGFR/ALK-negative/unselected NSCLC. The value of surgery should also be emphasized. The result should be further confirmed by RCTs.

## 1 Introduction

Lung cancer is the leading cause of cancer-related death worldwide, and non-small cell lung cancer (NSCLC) represents approximately 85% of lung cancer cases [1]. Around 25–30% of NSCLC patients develop brain metastases (BMs) [2]; moreover, NSCLC is the most common primary cancer that metastasizes to the brain [2]. The prognosis of NSCLC patients with BMs is dismal, with a median survival time of only approximately 1 month in the absence of treatment [3,4]. Several strategies have been employed to treat BMs from NSCLC, including surgery, targeted therapy, immune checkpoint therapy, chemotherapy, radiotherapy, and their combination [3,5]. Each of these treatments has both advantages and drawbacks, and their relative efficacies are not fully understood.

In recent years, targeted therapies for NSCLC and BMs have rapidly developed. For instance, the epidermal growth factor receptor (EGFR) plays an essential role in lung cancer and depends on its expression status among the population. The mutations in EGFR and its polymorphisms are associated with the onset of carcinogenesis, the prediction of the metastases, and the response to tyrosine kinase inhibitors (TKIs) [6,7]. EGFR mutations are detected in 15–35% of NSCLC, with a higher percentage observed in the Asian population than in Europeans [8–10]. For patients with EGFR mutations and BMs, previous studies have shown that third-generation EGFR–TKIs and EGFR–TKIs combined with chemotherapy or radiotherapy have favorable efficacy [11,12]. Anaplastic lymphoma kinase (ALK) rearrangement, which occurs in 2–7% NSCLC, is also a classic target [13]. ALK inhibitors (especially the second and third-generation inhibitors) have shown promising efficacy for NSCLC with BMs [14].

However, there are still a significant number of patients with negative EGFR/ALK NSCLC BMs. In addition, limited to socioeconomic factors, genomic tests cannot cover all the patients, which means the genomic status of many patients remains unknown. Therefore, treatments with broader indications (including surgery, radiotherapy, immune checkpoint therapy, chemotherapy, and other innovative therapies) are more suitable for such patients [15]. Surgery is recommended for BMs that are large, have significant perilesional edema, and result in neurological deficits. It can provide immediate relief from symptomatic mass effects and help to confirm the diagnosis [10]. Radiotherapy, which mainly consists of whole-brain radiation therapy (WBRT) and stereotactic radiosurgery (SRS), is considered the cornerstone of the treatment for BMs [10,16]. WBRT has previously been the standard treatment for BMs. However, considering the neurocognitive toxicity, the value of WBRT has been challenged, and SRS has gradually become popular [10,17,18]. As an alternative to surgical resection, SRS is a high precise localized irradiation given in one fraction. It can achieve a dose to the tumor with a low risk of damage to the surrounding normal brain [16]. SRS is recommended for BMs of a limited number (up to 4) and limited size (up to 3 cm) [18–20]. Immune checkpoint inhibitors (ICIs) represent a major breakthrough for treating metastatic NSCLC and have shown preliminarily promising outcomes in patients with BMs from NSCLC [10]. Nevertheless, previous studies generally analyzed single-arm treatments or compared pairwise treatments and could not generate clear hierarchies of treatment approaches. Consequently, we performed a systematic literature review and Bayesian network meta-analysis (NMA) to analyze the comparative efficacy of all types of therapy available for these patients.

## 2 Methods

### 2.1 Data sources and search strategy

This research was performed following the guidelines provided by the PRISMA (preferred reporting items for systematic reviews and meta-analyses) report [21] (PRISMA Checklist). We searched the PubMed, EMBASE, Web of Science, Cochrane Library, and ClinicalTrials.gov databases from inception until April 10, 2022, without language restrictions, for randomized control trials (RCTs). The search took into account both medical subject headings and text words, using the main search terms “NSCLC,” “brain metastasis,” and terms specific to the different treatments. The detailed search strategy for the databases is presented in Table S1. The reference lists of the relevant articles were checked for additional articles. The protocol was registered in the International Prospective Register of Systematic Reviews (PROSPERO, CRD42021227078).


**Ethical approval:** The conducted research is not related to either human or animal use.

### 2.2 Study selection

Two investigators, Zhang C.K. and Zhou W.J.L., independently assessed the eligibility of studies based on the title, abstracts, and full texts, resolving disagreements by obtaining a consensus with Guan X.D. We included published and unpublished trials that met the following criteria:

- Population: Adult (≥18 years) histologically or cytologically diagnosed NSCLC patients with one or more BMs; EGFR/ALK status was negative or unselected. There was no restriction on PD-L1 expression. Eligible participants had a Karnofsky Performance Status ≥60 and stable systemic disease with adequate hematologic, renal, and hepatic function.
- Interventions and comparisons: Two or more different arms of treatment for brain metastasis originating from NSCLC. ICIs were classified as anti-CTLA4 or anti-PD-(L)1 checkpoint inhibitors. Target therapy, such as EGFR-TKIs, was also included in our analyses if the participants were unselected for EGFR/ALK status. Chemotherapy referred to traditional NSCLC chemotherapy (platinum-based chemotherapy or other NSCLC chemotherapies recommended by NCCN guidelines). Other innovative medicines, such as temozolomide (TMZ, which is commonly used for glioma chemotherapy), were listed separately. Radiotherapy was defined as either WBRT, SRS, three-dimensional conformal radiation therapy (3D-CRT), or any of their combination.
- Outcomes: Central nervous system progression-free survival (CNS-PFS) or overall survival (OS) times were reported. Some of the included trials also reported the CNS objective response rate (ORR) according to Response Evaluation Criteria in Solid Tumors, version 1.1 (RECIST 1.1).
- Study design: RCTs were included. The follow-up period was required to be no shorter than 1 year. Since neurosurgical resection is an invasive and personalized treatment, it is impracticable to perform RCTs in terms of surgery. Therefore, retrospective trials comparing surgery or not were included in another separate meta-analysis.
- We excluded studies not adhering to the inclusion criteria. The other exclusion criteria were as follows:
- Studies only recruited patients NSCLC with EGFR-mutation or ALK-rearrangement.
- Trials comparing treatments that have not been approved by the US Food and Drug Administration.
- Reviews, animal experiments, basic research, case reports, and meta-analyses.

### 2.3 Data extraction and quality assessment

Two authors, Zhang C.K. and Zhou W.J.L., independently extracted data from the eligible studies and assessed the risk of bias in the individual studies. Disagreements were resolved by consensus or referral to a third reviewer, Guan X.D. The extracted items included study details (name of the first author, country, registration number, and phase of the study), participant details (number of participants, age, and gender), intervention and comparison in each arm, and survival outcomes (hazard ratios [HRs] and 95% confidence interval [CI], including the OS rate, PFS rate, and ORR).

The quality and risk of bias were assessed for each trial using the Cochrane Collaboration risk of bias tool [22], random sequence generation, allocation concealment, blinding of participants and personnel, blinding of outcome assessment, incomplete outcome data, selective outcome reporting, and other sources of bias were examined. The quality of each study was categorized as high, low, or unclear.

### 2.4 Statistical analyses

The risk of bias in the RCTs was assessed by Review Manager (RevMan, 5.3, The Cochrane Collaboration, London, UK). The Bayesian NMA was performed using the JAGS program and GEMTC package in R software (version 4.0.2, R Foundation, Vienna, Austria). HRs of CNS-PFS and OS rates were analyzed on a natural log scale and pooled as HRs and 95% credible intervals (CrIs). For studies that did not directly provide HRs, we extracted and estimated the HRs and corresponding standard error from a high-quality Kaplan–Meier curve with the methods described by Tierney [23]. The ORR was pooled with the risk ratio (RR) and corresponding 95% CrI. The simulation was performed using the Markov chain Monte Carlo technique with three different chains, and each of them produced 10,000 interactions with 100,000 burn-in samples and ten thinning rates. Fixed-effect models were used, since in most cases, the treatment of interest was evaluated in only one trial. We assessed statistical inconsistencies by the edge-splitting method to compare direct and indirect evidence. Statistical significance was considered when *P* < 0.05. Statistical heterogeneity was estimated by the I2 statistic, which indicates what proportion of variability in outcomes was due to heterogeneity rather than chance. An I2 > 50% was regarded as significant heterogeneity, while I2 < 25% indicated a small level of heterogeneity. To assess the robustness and reliability of the results, we also performed a sensitivity analysis in the absence of low-quality trials.

## 3 Results

### 3.1 Study selection and characteristics

**Figure 1:**
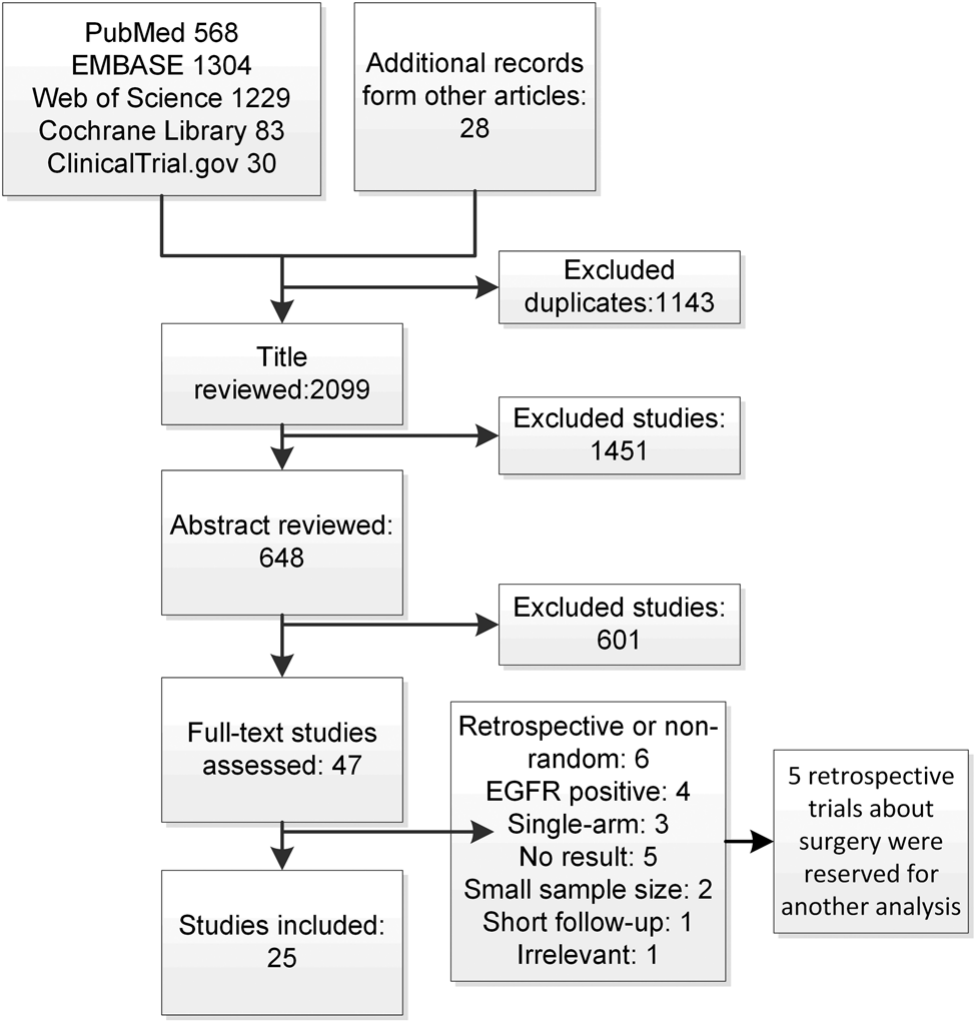
Flowchart of study selection. A total of 25 randomized controlled trials that met the inclusion criteria were included in the NMA.

The procedures of the screening and the reasons for exclusion are shown in Figure 1. A total of 2,099 studies met the search criteria. After title and abstract screening, 47 trials were retrieved, and the full text was reviewed. Ultimately, 25 trials [24–48] were included in this NMA. Another five trials were included in a traditional meta-analysis comparing surgery or not. As shown in Table 1, a total of 3,054 participants were enrolled in the selected RCTs. Most participants were male and over 55 years of age. The demographic and clinical characteristics kept a balance between the intervention and control groups in each RCT. The trial publication dates ranged from 2005 to 2022. The bias assessment is presented in Figure S1, with two trials assessed as having a high risk of bias [26,27] and 23 trials assessed as having a low risk of bias [24,25,28–48].

**Table 1:** Characteristics of included trials

Author/Year	Study number (Phase)	Country	No. of patients (I/C)	Males% (I/C)	Age^†^ (I/C)	Previous treatment for BMs	Intervention arm	Control arm
Lim et al. [24] 2015	NCT01301560 (III)	Korea	49/49	71%/73%	58 (33–77)/57 (29–85)	No	SRS + Chem	Chem
Zhu et al. [34] 2018	NA	China	31/31	55%/58%	NA	24/31 in I and 19/31 in C received Chem; 11/31 in I and 9/31 in C received targeted therapy	3D-CRT (18–36 Gy) + TMZ	CRT(18–36 Gy)
GlaxoSmithKline [28] 2006	NCT00390806(Ⅲ)	Multiple	236/236	64.4%/66.9%	59.4 ± 8.56/57.8 ± 8.65	Received chemotherapy	WBRT(30 Gy/10fra) + Chem	WBRT(30 Gy/10fra)
Sperduto et al. [25] 2013	NCT00096265(Ⅲ)	US and Canada	40/44	NA	63(NA)/64(NA)	No	WBRT(37.5 Gy/15fra) + SRS(15–24 Gy, size depended) + TMZ	WBRT(37.5 Gy/15fra) + SRS(15–24 Gy, size depended)
			41/44	NA	61(NA)/64(NA)	No	WBRT(37.5 Gy/15fra) + SRS(15–24 Gy, size depended) + Erlotinib	WBRT(37.5 Gy/15fra) + SRS(15–24 Gy, size depended)
He et al. [27] 2017	NCT02284490(Ⅱ)	China	32/28	34.4%/39.3%	59(52–73)/58.5(45–77)	No	WBRT(30 Gy/10fra) + Chem	WBRT(30 Gy/10fra) + TMZ
Hassler et al. [26] 2013	NCT00266812(Ⅱ)	Austrian	22/13	59%/61.5%	69(36–85)/64(54–78)	No	WBRT(40 Gy/20fra or 30 Gy/10fra) + TMZ	WBRT(40 Gy/20fra or 30 Gy/10fra)
Guerrieri [30] 2004	NA(Ⅲ)	Australia	21/21	71%/71%	60(42–77)/63(39–78)	No	WBRT(20 Gy/5fra) + Chem	WBRT(20 Gy/5fra)
Chua et al. [29] 2010	NCT00076856(Ⅱ)	China	47/48	64%/67%	59(38–78)/62(43–79)	No	WBRT(30 Gy/10fra,over 2 week) + TMZ	WBRT
Chabot et al. [31] 2017	NCT01657799(II)	Multiple	102/102	65%/55%	62(39–81)/60(41–86)	No	WBRT(30 Gy/10fra) + Veli	WBRT(30 Gy/10fra)
Lee et al. [35] 2014	NCT00554775(II)	Britain	40/40	37.5%/52.5%	61.3(48–75)/62.2(41–73)	No	WBRT(20 Gy/5fra) + Erlotinib	WBRT(20 Gy/5fra)
Grønberg et al. [37] 2012	NCT00415363(II)	Multiple	39/41	NA	NA	No	WBRT(20 Gy/5fra or 30 Gy/10fra) + Enza	WBRT(20 Gy/5fra or 30 Gy/10fra)
Jiang et al. [33] 2014	NCT01410370(II)	China	40/40	NA	NA	No	WBRT(30 Gy/10fra,2 week) + Endo	WBRT(30 Gy/10fra, 2 week)
Zhao et al. [32] 2016	NA(II)	China	40/40	52.5%/55%	67(57–75)/64(59–75)	No	WBRT(30 Gy/10fra,2 week) or 3D-CRT + Endo	WBRT(30 Gy/10fra, 2 week) or 3D-CRT
Yang et al. [36] 2020	NCT01887795(III)	China	106/114	59.4%/60.5%	55.5(26–70)/56(27–70)	No	WBRT(40 Gy/20fra) + Erlotinib	WBRT(40 Gy/20fra)
Pesce et al. [38] 2012	NCT00238251(III)	Switzerland	16/43	56.3%/62.8%	57(46–82)/63(45–79)	9/59 received Chem	WBRT(30 Gy/10fra) + Gefitinib	WBRT(30 Gy/10fra)
Wang et al. [40] 2015	NA(II)	China	37/36	67.6%/63.9%	61(NA)/62(NA)	No	3D-CRT(50 Gy) + Gefitinib	CRT(50 Gy) + Chem
Arrieta et al. [39] 2022	NCT04338867(II)	Mexico	46/50	54.3%/45.7%	61.4 ± 10.2/57.9 ± 12.8	No	WBRT(30 Gy/10fra) + Nitro	WBRT(30 Gy/10fra)
Gadgeel et al. [41] 2019	NCT02008227(Ⅲ)	Multiple	61/62	55.7%/53.2%	59.0(39–79)/62.5(39–83)	Previously treated and asymptomatic	Atez	Chem
Sezer et al. [48] 2021	NCT03088540(III)	Multiple	34/34	NA	NA	Previously treated and stable	Cemi	Chem
Borghaei et al. [42] 2015	NCT01673867(III)	Multiple	43/42	NA	NA	Previously treated, 74% had radiotherapy	Nivo	Chem
Wu et al. [43] 2019	NCT02613507(III)	China	45/27	NA	NA	Previously treated and stable	Nivo	Chem
Paz-Ares et al. [44] 2021	NCT03215706(III)	Multiple	64/58	NA	NA	Previously treated and stable	Nivo + Ipil	Chem
Hellmann et al. [45] 2019	NCT02477826(III)	Multiple	64/51	NA	NA	Previously treated and stable	Nivo + Ipil	Chem
Mansfield et al. [46] 2021	NCT01295827	Multiple	199/94	49.7%/56.4%	59.0(31–88)/60.0(31–81)	Previously treated and stable	Pemb	Chem
	NCT01905657							
	NCT02142738							
	NCT02220894							
Powell et al. [47] 2019	NCT02039674	Multiple	105/66	NA	NA	Previously treated and stable	Pemb + Chem	Chem
	NCT02578680							
	NCT02775435							

^†^Patients’ age was described as median age (range) or average age ± SD.

Abbreviations: BMs, brain metastases; I, intervention group; C, control group; SRS, stereotactic radiotherapy; WBRT, whole-brain radiotherapy; fra, fraction; 3D-CRT, three-dimensional conformal radiation therapy; TMZ, temozolomide; Chem, chemotherapy; Veli, veliparib; Enza, enzastaurin; Nitro, nitroglycerin; Endo, endostatin; Atez, atezolizumab; Cemi, cemiplimab; Nivo, nivolumab; Ipil, ipilimumab; and Pemb, pembrolizumab.

NA, not available.

Seventeen studies compared radiotherapy with radiotherapy plus systemic therapies [24–40]. Such systemic therapies include traditional chemotherapy for NSCLC, EGFR-TKIs (Gefitinib or Erlotinib), and six kinds of innovative therapies (TMZ, Endostar [Endo], Enzastaurin [Enza], Nitroglycerin [Nitro], and Veliparib [Veli]). Since ALK inhibitors were only applied to patients with ALK-positive NSCLC [14], relevant studies were not included in the current study about EGFR/ALK negative or unselected patients. These studies mainly recruited patients with newly diagnosed BMs, and the BMs have not previously received local treatment (neither surgery nor radiotherapy). Only three studies recruited some patients who had previously received systemic chemotherapy or targeted therapy [28,34,38]. In each included trial, the intervention and control groups applied the same radiation technique and dose, except for Lim’s study [24], which compared SRS + chemotherapy with chemotherapy alone. According to the current guideline, WBRT was recommended at a standard dose (30 Gy in ten fractions) or a lower dose (20 Gy in five fractions) for patients with newly diagnosed BMs [18]. For trials included in our analyses, eight trials performed WBRT of 30 Gy in ten fractions [27–39]; two trials performed WBRT of 20 Gy in five fractions [30,35]; and one trial performed WBRT of either 30 Gy in ten fractions or 20 Gy in five fractions [37]. In addition, several trials applied WBRT at a higher dose. Yang’s trial performed WBRT of 40 Gy in 20 fractions [36]. Hassler’s trial performed WBRT of either 30 Gy in ten fractions or 40 Gy in 20 fractions [26]. Two trials performed 3D-CRT of 18–36 Gy [34] or 50 Gy [40]. One trial performed both WBRT (37.5 Gy in 15 fractions) and size-dependent SRS (lesions <2 cm, 2.1–3.0 cm, and 3.1–4.0 cm received 24, 18, and 15 Gy, respectively) [25]. Only one trial performed SRS with an unclear dose [24]. Trials on Nitro, Veli, Enza, and Endo performed concurrent systemic therapy with radiotherapy [31–33,37,39]. Trials on EGFR-TKI, TMZ, and traditional chemotherapy performed systemic therapy both during and after radiotherapy [25–30,34–36,38,40]. One trial started chemotherapy within 3 weeks after SRS [24].

Eight studies compared ICI-based therapies with chemotherapy, including Pembrolizumab (Pemb), Atezolizumab (Atez), Cemiplimab (Cemi), Nivolumab (Nivo), and Ipilimumab (Ipil). These eight studies recruited patients with previously treated (radiation therapy and/or surgery for BMs) and clinically stable BMs [41–48].

In addition, five retrospective studies [49–53] compared surgery (*n* = 269) with radiotherapy alone (*n* = 431) for patients who have opportunities for surgery (Table S2). Seven hundred previously untreated resectable BMs from NSCLC were included in analyses.

Considering the heterogeneity of the study design and treatment history, we divided the analyses into three parts: (1) NMA about radiotherapy or systemic therapy for previously untreated patients; (2) NMA about ICIs or chemotherapies for previously treated patients; and (3) traditional meta-analysis comparing surgery with radiotherapy alone for patients who had opportunities of surgery.

### 3.2 OS

**Figure 2:**
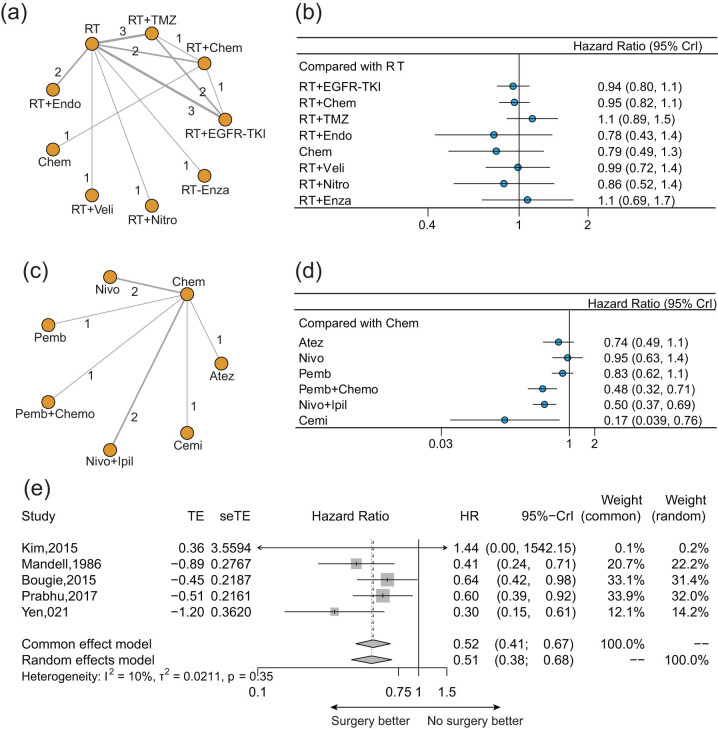
Pooled result of OS for different treatments for BMs from EGFR/ALK-negative/unselected NSCLC. (a) Network diagram and (b) forest plot of OS for different treatments compared with radiotherapy alone in newly diagnosed BMs. (c) Network diagrams and (d) forest plot of OS for different treatments compared with chemotherapy alone in previously treated BMs. (e) Forest plot of OS comparing surgery with radiotherapy alone. Each node in the network diagram represents one treatment, and the numbers represent direct head-to-head comparisons. Abbreviations: RT, radiotherapy; TMZ, temozolomide; Chem, chemotherapy; EGFR-TKI, epidermal growth factor receptor-tyrosine kinase inhibitors; Enza, enzastaurin; Nitro, nitroglycerin; Veli, veliparib; Endo, endostatin; Atez, atezolizumab; Cemi, cemiplimab; Nivo, nivolumab; Ipil, ipilimumab; and Pemb, pembrolizumab.

The NMA results for the OS outcome are displayed in Figure 2. Sixteen radiotherapy-related trials about nine regimens reported OS for previously untreated BMs (Figure 2a). Unfortunately, none of such regimens showed a significant survival benefit over radiotherapy alone (Figure 2b). Radiotherapy + endostatin (HR: 0.78, 95% CrI: 0.43–1.4), radiotherapy + nitroglycerin (HR: 0.86, 95% CrI: 0.52–1.4), and chemotherapy alone (HR: 0.79, 95% CrI: 0.49–1.3) showed slightly beneficial trends on OS without statistical significance. Radiotherapy combined with EGFR-TKIs, or other innovative medicines (TMZ, Veli, or Enza) had similar or even worse effects than radiotherapy alone in terms of OS.

Eight studies compared six kinds of ICI-based treatments with chemotherapy for previously treated BMs reported OS (Figure 2c). Pembrolizumab + chemotherapy (HR: 0.48, 95% CrI: 0.32–0.71), nivolumab + ipilimumab (HR: 0.50, 95% CrI: 0.37–0.69), and cemiplimab (HR: 0.17, 95% CrI: 0.039–0.76) significantly prolonged OS than chemotherapy (all *P* < 0.05) (Figure 2d). Atezolizumab monotherapy (HR: 0.74, 95% CrI: 0.49–1.1) and pembrolizumab monotherapy (HR: 0.83, 95% CrI: 0.62–1.1) also showed beneficial trend than chemotherapy in terms of OS (all *P* > 0.05). Nivolumab monotherapy had similar effect than chemotherapy on OS (HR: 0.95, 95% CrI: 0.63–1.4). The derived HR for treatments compared with each other are demonstrated in Figure S2.

Five trials compared OS for patients who performed surgery or not (Figure 2e). Surgical resection of BMs from NSCLC was associated with a significantly favorable OS than radiotherapy alone (HR: 0.52, 95% CrI: 0.41–0.67, common effect model).

### 3.3 CNS-PFS

**Figure 3:**
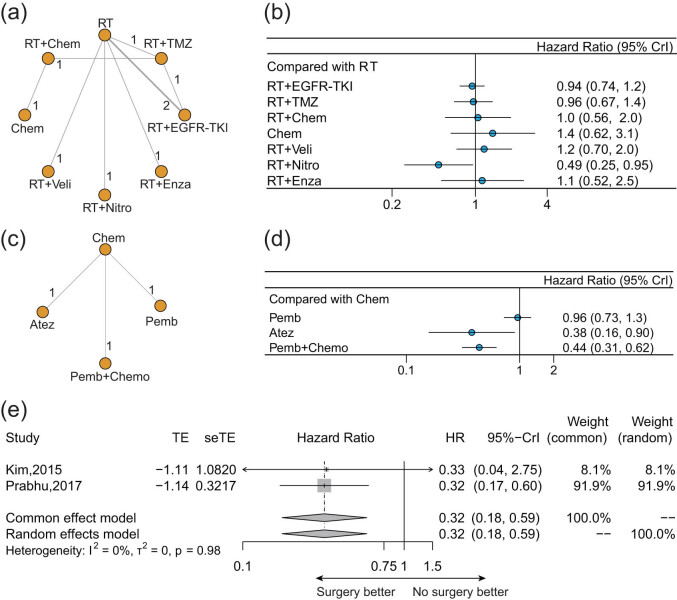
Pooled result of CNS-PFS for different treatments for BMs from EGFR/ALK-negative/unselected NSCLC. (a) Network diagram and (b) forest plot of CNS-PFS for different treatments compared with radiotherapy alone in newly diagnosed BMs. (c) Network diagrams and (d) forest plot of CNS-PFS for different treatments compared with chemotherapy alone in previously treated BMs. (e) Forest plot of CNS-PFS comparing surgery with radiotherapy alone. Each node in the network diagram represents one treatment, and the numbers represent direct head-to-head comparisons. Abbreviations: RT, radiotherapy; TMZ, temozolomide; EGFR-TKI, epidermal growth factor receptor-tyrosine kinase inhibitors; Enza, enzastaurin; Nitro, nitroglycerin; Veli, veliparib; Chem, chemotherapy; Pemb, pembrolizumab; and Atez, atezolizumab.

As shown in Figure 3a, nine studies reported CNS-PFS in terms of eight kinds of radiotherapy-associated treatments for previously untreated BMs: radiotherapy alone, radiotherapy + EGFR-TKI, radiotherapy + chemotherapy, chemotherapy alone, and radiotherapy combined with other innovative systemic agents (TMZ, Nitro, Enza, or Veli). Only radiotherapy + nitroglycerin showed significant benefit over radiotherapy in terms of CNS-PFS (HR: 0.49, 95% CrI: 0.25–0.95) (Figure 3b). Nevertheless, compared with radiotherapy alone, radiotherapy + EGFR-TKIs, radiotherapy + chemotherapy, and radiotherapy combined with other innovative medicines (TMZ, Enza, or Veli) did not derive significant survival benefits for CNS-PFS (all *P* > 0.05) (Figure 2b).

Regarding ICIs for previously treated BMs, three trials about four treatments reported CNS-PFS and were included in analyses (Figure 3c). Atezolizumab (HR: 0.38, 95% CrI: 0.16–0.90), pembrolizumab + chemotherapy (HR: 0.44, 95% CrI: 0.31–0.62) showed statistically significant benefits than chemotherapy alone on CNS-PFS (Figure 3d). Pembrolizumab monotherapy showed a similar effect with chemotherapy on CNS-PFS (HR: 0.96, 95% CrI: 0.73–1.3).

Only two trials reported CNS-PFS in terms of surgery (Figure 3e). Surgical resection of BMs from NSCLC still showed better CNS-PFS than radiotherapy alone (HR: 0.32, 95% CrI: 0.18–0.95, common effect model).

### 3.4 ORR

**Figure 4:**
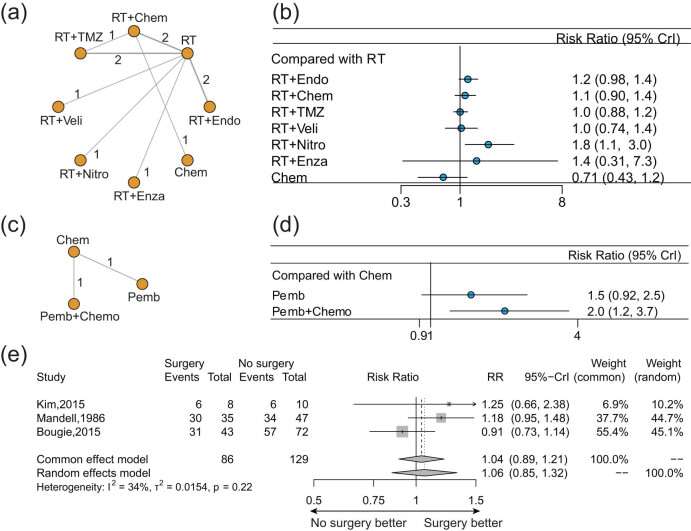
Pooled result of ORR for different treatments for BMs from EGFR/ALK-negative/unselected NSCLC. (a) Network diagram and (b) forest plot of ORR for different treatments compared with radiotherapy alone in newly diagnosed BMs. (c) Network diagrams and (d) forest plot of ORR for different treatments compared with chemotherapy alone in previously treated BMs. (e) Forest plot of ORR comparing surgery with radiotherapy alone. Each node in the network diagram represents one treatment, and the numbers represent direct head-to-head comparisons. Abbreviations: RT, radiotherapy; TMZ, temozolomide; Chem, chemotherapy; Endo, endostatin; Enza, enzastaurin; Nitro, nitroglycerin; Veli, veliparib; and Pemb, pembrolizumab.

Eleven radiotherapy-related trials about eight kinds of treatments reported ORR (Figure 4a). Radiotherapy + nitroglycerin showed a significant benefit over radiotherapy alone on ORR (RR: 1.8, 95% CrI: 1.1–3.0). Adding chemotherapy or other innovative medicines (TMZ, Endo, Veli, or Enza) to radiotherapy did not show significant benefit over radiotherapy alone (all *P* > 0.05). Patients treated with chemotherapy alone had the lowest ORR (versus radiotherapy RR: 0.71, 95% CrI: 0.43–1.2) (Figure 4b).

Only two trials about ICIs for previously treated BMs reported ORR (Figure 4c). Pembrolizumab + chemotherapy showed significantly better ORR than chemotherapy (RR: 2.0, 95% CrI: 1.2–3.7), while pembrolizumab monotherapy did not (RR: 1.5, 95% CrI: 0.92–2.5) (Figure 4d).

Three trials about surgery reported ORR (Figure 4e). Surgery showed a favorable trend of ORR for BMs from NSCLC but did not reach a statistical difference (RR: 1.04, 95% CrI: 0.89–1.21, common effect model).

### 3.5 Heterogeneity, consistency, and sensitivity analysis

There was low global heterogeneity for the comparison of radiotherapy-associated regiments (I2 = 0% for OS, I2 = 0% for CNS-PFS, and I2 = 8% for ORR) and low to moderate heterogeneity for the comparison of ICIs (I2 = 22% for OS, I2 = 33% for CNS-PFS, and I2 = 25% for ORR). Furthermore, local heterogeneities were also acceptable between paired treatments (Table S3). In terms of inconsistency, there was no significant difference between direct and indirect comparisons of the OS, CNS-PFS, and ORR (Figure S3). During the sensitivity analysis, one study with unclear random sequence generation [27] and another study with imbalanced patients’ initial baseline [26] were excluded. The results showed the same ranks compared with those of the original NMA (Figure S4). The sensitivity analyses showed that the overall results remained robust.

## 4 Discussion

Currently, there is a wide range of alternative treatments available for brain-metastatic NSCLC with negative or unselected EGFR/ALK status. Nevertheless, direct comparisons of such treatments are limited. Our study analyzed the relative efficacy of each treatment for previously treated and untreated BMs. It showed that several ICIs were associated with longer OS and CNS-PFS than chemotherapy in patients with previously treated BMs. Except for nitroglycerin, the addition of EGFR-TKIs, chemotherapy, and other non-ICI systemic innovative medicines to RT did not improve OS, CNS-PFS, and OS. Surgery of BMs was associated with better OS, CNS-PFS, rather than ORR. The reasons for these findings are presented as follows.

Currently, ICIs have been the standard first-line treatments for metastatic NSCLC without sensitizing EGFR or ALK or other druggable mutations [54]. However, the intracranial efficacies of ICIs remained uncertain. The exact mechanism of ICIs for brain tumors was also unclear. First, it may be related to modified immune cell activity rather than direct action in the brain. By immune cell trafficking and T-cell priming in the extracranial immune system, ICIs could produce an effective immune response in the CNS [55,56]. Moreover, due to the infiltration of lymphocytes in BMs [57] and the relatively stable PD-L1 expression level between primary tumors and BMs [58], it can be hypothesized that PD-(L)1 inhibitors might provide similar effects inside and outside the brain [59].

It is not surprising that ICIs were associated with favorable efficacies. Current RCTs have confirmed that PD-(L)1 inhibitors could significantly improve OS and PFS rates among patients with metastatic NSCLC [60–63]. For NSCLC patients with BMs, the results of other concurrent studies are also consistent with our findings. The FIR study demonstrated that atezolizumab monotherapy showed clinical activity in NSCLC patients with or without BMs [64]. Thirteen patients with pretreated BMs receiving atezolizumab were enrolled, with an ORR of 23% (3/13) and a median PFS and OS interval of 4.3 months (95% CI: 1.1–16.2) and 6.8 months (95% CI: 3.2–19.5), respectively [64]. The KEYNOTE-001 clinical trial first confirmed the efficacy of pembrolizumab in NSCLC patients with or without BMs [65]. Goldberg’s non-random phase II study showed that when treating BM originating from NSCLC with PD-L1 expression ≥1% with pembrolizumab, the ORR, median OS time, and median PFS time were 29.7% (11/37), 9.9 months (95% CI: 7.5–29.8), and 1.9 months (95% CI: 1.8–3.7), respectively [59]. A cohort of 409 patients with BMs from NSCLC showed that the median OS and disease control rate reached 8.6 months (95% CI: 6.4–10.8) and 40% (164/409) after applying nivolumab [66]. Kitadai also found a PD-L1-negative NSCLC patient with BMs reached intracranial complete response after nivolumab monotherapy [67].

In addition, the choice of monotherapy or combined therapy also affects the therapeutic effect of ICIs for BMs. In our NMA, several combined therapies, including pembrolizumab + chemotherapy and nivolumab + ipilimumab derived relatively better effects. Current NCCN guideline recommends ICIs monotherapy for patients with PD-L1-expression more than 50%; whereas, ICIs in combination with chemotherapy is recommended regardless of PD-L1 expression [54]. Consistently, comparisons between different immunotherapy strategies for BMs also showed that combined ICIs with chemotherapy or dual ICIs had favorable efficacies for advanced NSCLC [57,68,69]. Similarly, an RCT also showed that nivolumab + ipilimumab derived better CNS-PFS and ORR than nivolumab monotherapy for BMs from melanoma [70].

Moreover, the included RCTs were aimed at previously treated BMs, whereas the efficacy of ICIs + radiotherapy for untreated BMs lacked evaluation. A meta-analysis of 19 prospective or retrospective studies showed that ICIs + radiotherapy could significantly prolong OS than RT alone (HR: 0.77, 95% CI:0.71–0.83) for BMs from NSCLC, and grade 3–4 neurological adverse event rates were similar (RR: 0.91, 95% CI:0.41–2.02) [71]. Recently, a retrospective study of 21 patients with BMs from EGFR/ALK-negative NSCLC also found the concurrent WBRT and ICIs prolonged CNS-PFS (HR: 0.29, 95% CI: 0.11–0.80; *P* = 0.016) and OS (HR 0.33, 95% CI: 0.08–1.12; *P* = 0.107) than WBRT alone [72].

The expression level of PD-L1 could also affect the response to ICIs. Studies have demonstrated that ICIs have better efficacy in NSCLC (with or without BM) patients whose PD-L1 expression is ≥1% [59,65,73], while responses can still occur in those with PD-L1 expression <1% or PD-L1-negative tumors [44,67,73–75]. Of the eight trials included in this study, six trials did not select patients according to PD-L1 expression [41–45,47], and ICIs still showed promising efficacies. One trial about pembrolizumab monotherapy recruited patients with PD-L1 expression of no less than 1%, but its effect was still inferior to pembrolizumab + chemotherapy [46]. Another trial about cemiplimab included patients with PD-L1 expression at least 50%, and showed relatively superior OS and CNS-PFS [48]. Therefore, we speculated that the therapeutic effect was determined by both PD-L1 expression and the properties of ICIs.

Nowadays, radiotherapy (SRS or WBRT) remains the mainstay of initial therapy for BMs [16]. Previous studies have shown the addition of WBRT to SRS or surgery alone could increase CNS-PFS and local control rate; however, the OS time did not prolong, and the neurocognitive toxicity also increased [18,76,77]. Therefore, local treatment (SRS or surgical resection) without WBRT is recommended for patients with up to four BMs and good physical performance [18]. With the development of the SRS technique, SRS was tried to treat selected patients with multiple (more than four) BMs. Several multicenter studies have found that patients treated with SRS for 5–10 BMs, or even 5–15 BMs derived comparable OS to those with 2–4 BMs [78,79]. WBRT is often considered for patients who are not suitable for SRS or surgery (e.g., innumerable metastases, innumerable metastases, poor physical performance, or other contraindications) [10], and was believed to prolong CNS-PFS [18]. Nevertheless, an RCT found that the WBRT showed no difference with optimal supportive care in terms of OS, quality of life, and dexamethasone for patients unsuitable for resection or SRS [80].

In current analyses, nitroglycerin + WBRT showed favorable effects for BMs. Nitroglycerin has just been used to assist tumor radiotherapy in recent years. It could reduce the radiation resistance by alleviating tumor hypoxia [39]. Nevertheless, the synergistic effect of nitroglycerin with chemoradiotherapy was only tested in several phase II trials of primary NSCLC [81–83] and only one trial about BM [39], and the results of primary NSCLC were controversial [81–83]. Therefore, the efficacy of nitroglycerin on BMs needs to be further evaluated.

On the other hand, there was no significant advantage of adding chemotherapy, EGFR-TKI, or other non-ICI innovative systemic agents to radiotherapy. Systematic reviews have revealed that radiotherapy + chemotherapy might improve response rates compared with radiotherapy alone; however, this approach does not improve survival outcomes and increases the incidence of adverse reactions in patients with BMs arising from lung cancer [84,85]. Meanwhile, WBRT plus systemic therapy was associated with increased risks for vomiting compared to WBRT alone [84,86–88]. For the same reasons, the CNS (Congress of Neurological Surgeons) and EANO (European Association of Neuro-Oncology) guidelines did not suggest routine use of cytotoxic chemotherapy either alone or following WBRT [16,89].

Although TMZ is recommended to be used with WBRT for patients with BMs arising from triple-negative breast cancer [89], its efficacy on BM arising from NSCLC is controversial. Several trials have yielded mixed results [90–93], while the current systematic review and meta-analysis determined that adding TMZ to radiotherapy can increase the ORR [87,94,95]. However, it is generally believed that adding TMZ cannot induce a better OS outcome [86,87,96,97]. Therefore, there is insufficient evidence to conclude that there is value in adding TMZ for the treatment of NSCLC with BM [98].

Although EGFR-TKIs were used to treat BMs from EGFR-unselected NSCLC, the effect was not ideal. Currently, with the widespread use of genetic testing technology, it is recommended to screen for EGFR-mutations in NSCLC patients with BMs and treat them with third-generation EGFR-TKIs, which have better blood–brain barrier penetrability and better efficacy [99].

Adding several other innovative systemic treatments to radiotherapy did not show survival benefits, including Veli (polyadenosine-diphosphate-ribose polymerase inhibitor), Enza (serine/threonine kinase inhibitor), and Endo (an antiangiogenic drug). This was not surprising because RCTs evaluating treatments for NSCLC (with or without metastases) have demonstrated that although Veli [100] or Endo [101] demonstrated a favorable trend in PFS and OS outcomes versus chemotherapy alone, the differences were not statistically significant; however, adding Enza to chemotherapy may induce shorter median survival times [102].

Surgical resection of BMs remains one of the mainstays of therapies for patients with BMs from NSCLC [103]. Since these metastases show radioresistance compared to SCLC, surgical resection to relieve the space-occupying effect is often the first step in treatments for these patients [10]. In the current analyses, surgery could derive better OS and CNS-PFS than radiotherapy alone for BMs from NSCLC. Consistently, previous studies also found surgery improved survival outcomes of patients with a single brain-metastatic lesion, a good karnofsky performance scale (KPS), and a limited number of extracranial metastases (primary malignancies were not filtered) [104,105].

## 5 Limitations

There were several limitations in this study. First, the exact techniques of RT were not compared separately. On the one hand, the indications and efficacies of WBRT and SRS have been proven by high-quality studies. On the other hand, network comparisons could not form if radiotherapy techniques were discussed separately. Therefore, our analyses mainly focused on the effect of adjuvant systemic therapy on radiotherapy. Discuss RT as a whole is feasible and consistent with previous meta-analyses on BMs [106,107], because the intervention (with adjuvant systemic therapy) and control (without adjuvant systemic therapy) groups in each trial have received radiotherapy of the same technique and dose.

There was still potential selection and publication bias. First, as mentioned above, two trials had unqualified patient inclusion processes; therefore, a sensitivity analysis was conducted, and relatively robust results were ensured. Second, there were a limited number of trials in each comparison. For this reason, we did not use funnel plots to assess publication bias or small-study effects. Third, we assumed that the patients from different trials were similar; however, patients may have had different baseline levels. For example, an unbalanced baseline can arise from the presence or absence of symptoms and the exact number and volume of BMs. All of these factors could give rise to a bias.

Moreover, limited information also restricted our analyses. We could not analyze the adverse effects and quality of life (such as KPS score and other parameters), because such information was not available or not complete. Trials on systemic therapy or radiotherapy usually did not report how many patients underwent surgery and their corresponding outcome. Therefore, we could not analyze the synergy between surgery and other treatment. Included trials about surgery did not perform subgroup analysis according to the surgery details (such as location, size, and the number of BMs), which makes it difficult for us to obtain the corresponding summary results. In addition, most trials about ICIs included in our analyses only reported OS without reporting CNS-PFS and ORR. Such data of interest need to be further explored.

## 6 Conclusion

ICI-based therapies, especially ICI-combined therapies, showed promising efficacies for previously treated BMs from EGFR/ALK-negative/unselected NSCLC. Adding chemotherapy, EGFR-TKIs, and some innovative agents (TMZ, Nitro, Endo, Enza, and Veli) to radiotherapy showed limited effects than radiotherapy alone for newly diagnosed BMs from EGFR/ALK-negative/unselected NSCLC. Surgery could significantly prolong OS and CNS-PFS compared with radiotherapy alone. Limited to heterogeneity and available information, the result of the current network meta-analyses should be further confirmed by RCTs.

## Supplementary Material

Supplementary Material
