# Supplementary material for: Treatments for brain metastases from EGFR/ALK-negative/unselected NSCLC: A network meta-analysis
Source: Open Med (Wars). 2023 Feb 14;18(1):20220574. doi: 10.1515/med-2022-0574 (PMC9938645; doi:10.1515/med-2022-0574)
Supplement: Supplementary Material [file med-2022-0574-sm.pdf]

Supplementary material

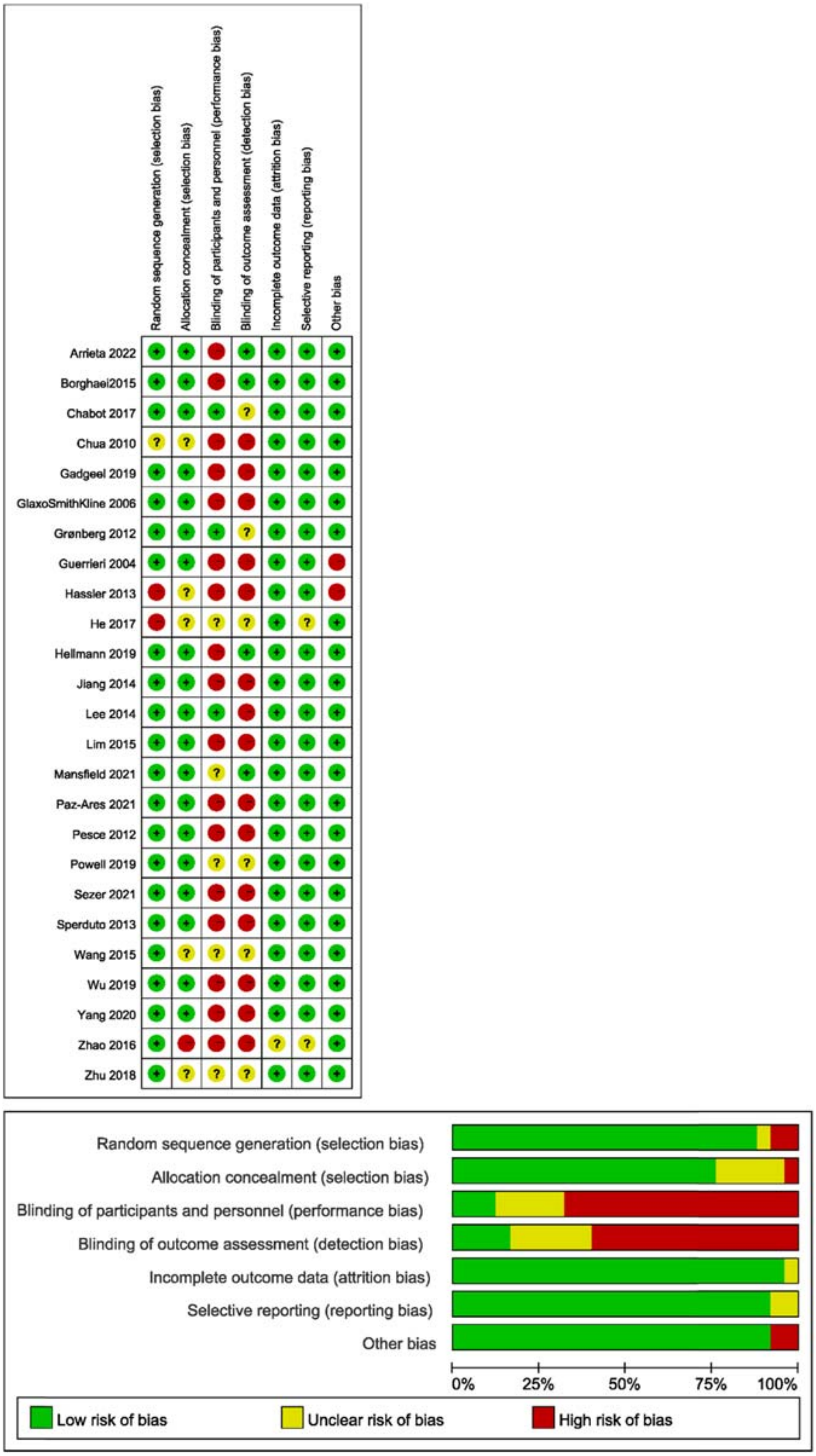

Figure S1: Risk of bias for included trials. +/green” = Yes; “-/red” = No; “?/yellow” = Unclear.

HR of OS for radiotherapy associated regimens

RT+EGFR-TKI	1.01 (0.88, 1.17)	1.21 (0.93, 1.58)	1.06 (0.9, 1.25)	0.83 (0.45, 1.51)	0.85 (0.53, 1.35)	1.05 (0.73, 1.51)	0.92 (0.54, 1.55)	1.16 (0.71, 1.87)
0.99 (0.86, 1.14)	RT+Chem	1.2 (0.92, 1.56)	1.05 (0.9, 1.21)	0.81 (0.45, 1.48)	0.83 (0.54, 1.31)	1.03 (0.73, 1.47)	0.9 (0.53, 1.53)	1.14 (0.7, 1.85)
0.82 (0.63, 1.08)	0.83 (0.64, 1.09)	RT+TMZ	0.87 (0.68, 1.13)	0.68 (0.36, 1.28)	0.7 (0.42, 1.17)	0.86 (0.57, 1.3)	0.75 (0.43, 1.32)	0.95 (0.56, 1.62)
0.94 (0.8, 1.11)	0.95 (0.83, 1.11)	1.14 (0.89, 1.47)	RT	0.78 (0.44, 1.39)	0.8 (0.5, 1.28)	0.99 (0.71, 1.36)	0.86 (0.52, 1.43)	1.09 (0.69, 1.73)
1.21 (0.66, 2.21)	1.23 (0.67, 2.24)	1.47 (0.78, 2.77)	1.29 (0.72, 2.29)	RT+Endo	1.02 (0.49, 2.18)	1.27 (0.65, 2.45)	1.11 (0.51, 2.39)	1.4 (0.67, 2.94)
1.18 (0.74, 1.89)	1.2 (0.76, 1.87)	1.44 (0.85, 2.41)	1.26 (0.78, 2)	0.98 (0.46, 2.05)	Chem	1.24 (0.7, 2.2)	1.08 (0.54, 2.17)	1.37 (0.71, 2.63)
0.95 (0.66, 1.37)	0.97 (0.68, 1.38)	1.16 (0.77, 1.75)	1.01 (0.73, 1.4)	0.79 (0.41, 1.54)	0.81 (0.45, 1.43)	RT+Veli	0.87 (0.48, 1.6)	1.11 (0.63, 1.93)
1.09 (0.64, 1.86)	1.11 (0.65, 1.88)	1.33 (0.76, 2.33)	1.16 (0.7, 1.92)	0.9 (0.42, 1.96)	0.93 (0.46, 1.85)	1.14 (0.63, 2.08)	RT+Nitro	1.27 (0.63, 2.52)
0.86 (0.53, 1.4)	0.87 (0.54, 1.43)	1.05 (0.62, 1.78)	0.92 (0.58, 1.45)	0.71 (0.34, 1.5)	0.73 (0.38, 1.42)	0.9 (0.52, 1.6)	0.79 (0.4, 1.58)	RT+Enza

HR of OS for immune checkpoint inhibitors

Atez	1.35 (0.89, 2.05)	1.29 (0.71, 2.33)	1.12 (0.67, 1.87)	0.65 (0.37, 1.16)	0.68 (0.41, 1.14)	0.23 (0.05, 1.05)
0.74 (0.49, 1.12)	Chem	0.95 (0.63, 1.44)	0.83 (0.62, 1.11)	0.48 (0.33, 0.71)	0.5 (0.37, 0.69)	0.17 (0.04, 0.73)
0.78 (0.43, 1.41)	1.05 (0.69, 1.59)	Nivo	0.87 (0.53, 1.45)	0.51 (0.29, 0.9)	0.53 (0.32, 0.89)	0.18 (0.04, 0.81)
0.89 (0.53, 1.48)	1.21 (0.9, 1.61)	1.14 (0.69, 1.9)	Pemb	0.58 (0.35, 0.94)	0.61 (0.4, 0.93)	0.21 (0.05, 0.9)
1.54 (0.86, 2.73)	2.08 (1.4, 3.07)	1.97 (1.12, 3.5)	1.73 (1.06, 2.82)	Pemb+Chemo	1.05 (0.63, 1.73)	0.36 (0.08, 1.6)
1.46 (0.88, 2.45)	1.98 (1.46, 2.7)	1.88 (1.13, 3.15)	1.64 (1.08, 2.52)	0.95 (0.58, 1.58)	Nivo+Ipil	0.34 (0.08, 1.49)
4.32 (0.96, 19.8)	5.82 (1.37, 25.46)	5.53 (1.23, 25.17)	4.83 (1.11, 21.59)	2.8 (0.62, 13.01)	2.94 (0.67, 13.3)	Cemi

HR of CNS-PFS for radiotherapy associated regimens

RT+EGFR-TKI	1.03 (0.7, 1.5)	1.07 (0.85, 1.35)	1.12 (0.6, 2.12)	1.49 (0.66, 3.34)	1.26 (0.71, 2.22)	0.52 (0.26, 1.04)	1.22 (0.53, 2.78)
0.97 (0.67, 1.42)	RT+TMZ	1.04 (0.72, 1.5)	1.09 (0.66, 1.82)	1.45 (0.7, 2.95)	1.23 (0.64, 2.34)	0.51 (0.24, 1.07)	1.19 (0.5, 2.82)
0.94 (0.74, 1.18)	0.96 (0.67, 1.39)	RT	1.05 (0.56, 1.96)	1.4 (0.63, 3.13)	1.18 (0.7, 2.01)	0.49 (0.25, 0.94)	1.14 (0.51, 2.51)
0.89 (0.47, 1.67)	0.92 (0.55, 1.51)	0.95 (0.51, 1.77)	RT+Chem	1.33 (0.8, 2.23)	1.12 (0.5, 2.58)	0.47 (0.19, 1.15)	1.09 (0.4, 2.95)
0.67 (0.3, 1.51)	0.69 (0.34, 1.42)	0.71 (0.32, 1.6)	0.75 (0.45, 1.25)	Chem	0.85 (0.32, 2.2)	0.35 (0.12, 0.98)	0.81 (0.27, 2.52)
0.79 (0.45, 1.41)	0.81 (0.43, 1.56)	0.85 (0.5, 1.43)	0.89 (0.39, 2.01)	1.18 (0.45, 3.09)	RT+Veli	0.41 (0.18, 0.95)	0.97 (0.37, 2.52)
1.92 (0.96, 3.84)	1.97 (0.93, 4.17)	2.05 (1.06, 3.96)	2.15 (0.87, 5.29)	2.86 (1.02, 8)	2.41 (1.05, 5.6)	RT+Nitro	2.34 (0.84, 6.48)
0.82 (0.36, 1.87)	0.84 (0.35, 2.02)	0.87 (0.4, 1.95)	0.92 (0.34, 2.48)	1.23 (0.4, 3.75)	1.03 (0.4, 2.7)	0.43 (0.15, 1.2)	RT+Enza

HR of CNS-PFS for immune checkpoint inhibitors

Pemb	1.04 (0.8, 1.36)	0.4 (0.16, 0.99)	0.46 (0.3, 0.72)
0.96 (0.73, 1.26)	Chem	0.38 (0.16, 0.9)	0.44 (0.31, 0.62)
2.52 (1.01, 6.23)	2.63 (1.11, 6.24)	Atez	1.16 (0.45, 2.92)
2.18 (1.4, 3.36)	2.27 (1.6, 3.2)	0.86 (0.34, 2.2)	Pemb+Chemo

RR of ORR for radiotherapy associated regimens

RT+Endo	0.85 (0.7, 1.02)	0.94 (0.71, 1.24)	0.85 (0.67, 1.08)	0.87 (0.59, 1.27)	1.51 (0.91, 2.61)	3.26 (0.38, 84.54)	0.6 (0.35, 1.01)
1.18 (0.98, 1.43)	RT	1.11 (0.9, 1.37)	1 (0.88, 1.17)	1.02 (0.74, 1.42)	1.77 (1.11, 2.98)	3.84 (0.45, 98.9)	0.71 (0.42, 1.15)
1.06 (0.8, 1.41)	0.9 (0.73, 1.11)	RT+Chem	0.9 (0.72, 1.12)	0.92 (0.63, 1.35)	1.6 (0.96, 2.79)	3.46 (0.4, 88.64)	0.64 (0.4, 0.98)
1.17 (0.93, 1.49)	1 (0.86, 1.14)	1.11 (0.89, 1.39)	RT+TMZ	1.02 (0.71, 1.46)	1.77 (1.09, 3.01)	3.82 (0.45, 98.7)	0.71 (0.42, 1.16)
1.15 (0.79, 1.68)	0.98 (0.7, 1.36)	1.08 (0.74, 1.6)	0.98 (0.68, 1.4)	RT+Veli	1.74 (0.98, 3.16)	3.75 (0.43, 99.34)	0.69 (0.38, 1.24)
0.66 (0.38, 1.1)	0.56 (0.34, 0.9)	0.62 (0.36, 1.04)	0.56 (0.33, 0.92)	0.58 (0.32, 1.02)	RT+Nitro	2.16 (0.24, 59.15)	0.4 (0.19, 0.78)
0.31 (0.01, 2.64)	0.26 (0.01, 2.21)	0.29 (0.01, 2.5)	0.26 (0.01, 2.23)	0.27 (0.01, 2.31)	0.46 (0.02, 4.21)	RT+Enza	0.18 (0.01, 1.64)
1.66 (0.99, 2.89)	1.41 (0.87, 2.37)	1.56 (1.02, 2.52)	1.41 (0.86, 2.39)	1.45 (0.81, 2.66)	2.52 (1.28, 5.18)	5.49 (0.61, 150.8)	Chem

RR of ORR for immune checkpoint inhibitors

Pemb	0.68 (0.4, 1.09)	1.38 (0.66, 2.93)
1.47 (0.92, 2.5)	Chem	2.02 (1.21, 3.66)
0.73 (0.34, 1.51)	0.49 (0.27, 0.82)	Pemb+Chemo

Figure S2: Pooled estimates of the network meta-analysis. Data in each cell were hazard ratios or risk ratios (95% credible intervals) for the comparison of column-defining treatment versus row-defining treatment. Hazard ratios less than one favors column-defining treatment for OS and CNS-PFS. Risk ratios more than one favors column-defining treatment for ORR. Abbreviations: OS, overall survival; CNS-PFS, central nervous system progression-free survival; ORR, objective response rate; HR, hazard ratio; RR, risk ratio; RT, radiotherapy; EGFR-TKI, epidermal growth factor receptor-tyrosine kinase inhibitors; TMZ, temozolomide; Chem, chemotherapy; Veli, veliparib; Enza, enzastaurin; Nitro, nitroglycerin; Endo, endostatin; Atez, atezolizumab; Cemi, cemiplimab; Nivo, nivolumab; Ipil, ipilimumab; Pemb, pembrolizumab.

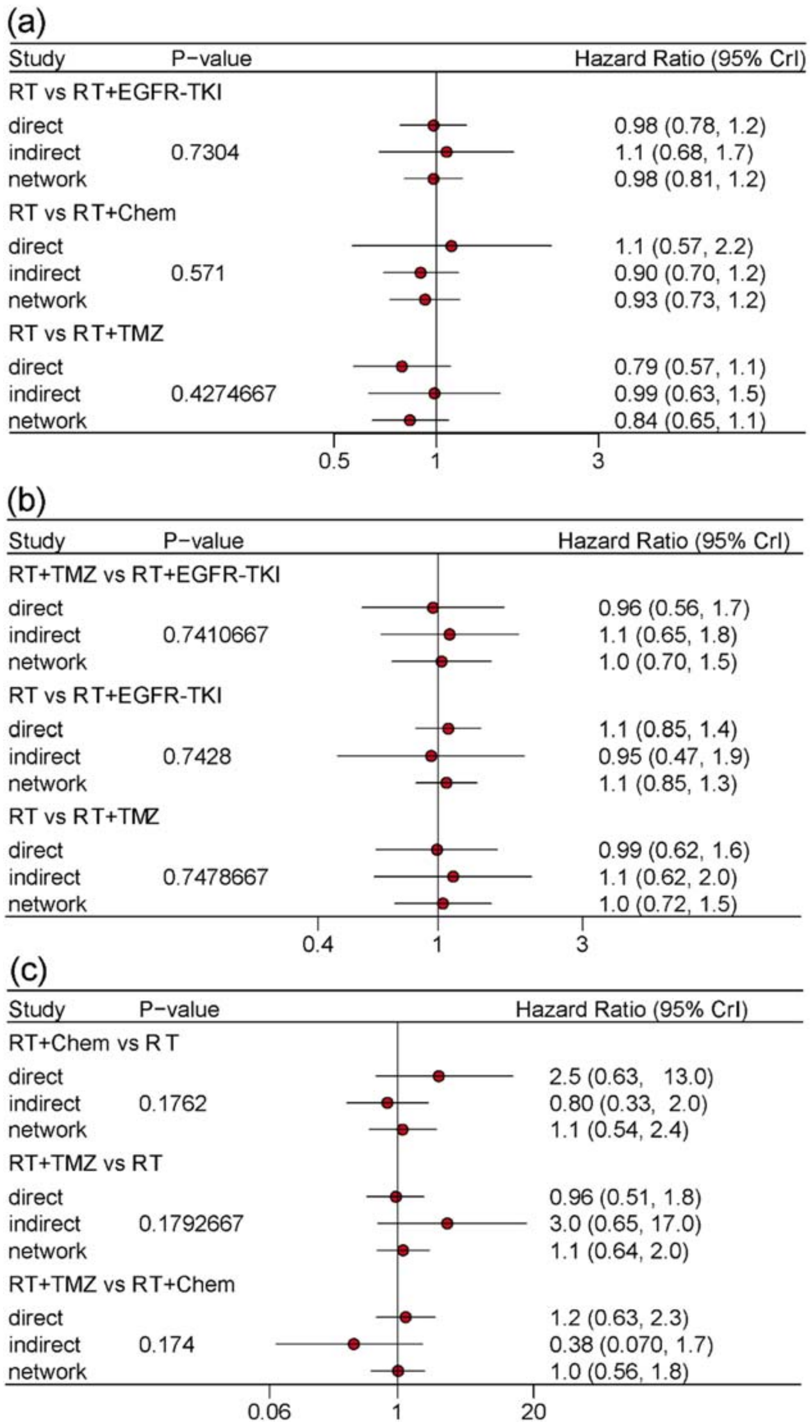

Figure S3: Inconsistency analyses for direct and indirect comparisons of the (a) OS, (b) CNS-PFS, and (c) ORR. A *P* value > 0.05 indicates no statistical inconsistencies between direct and indirect comparisons.

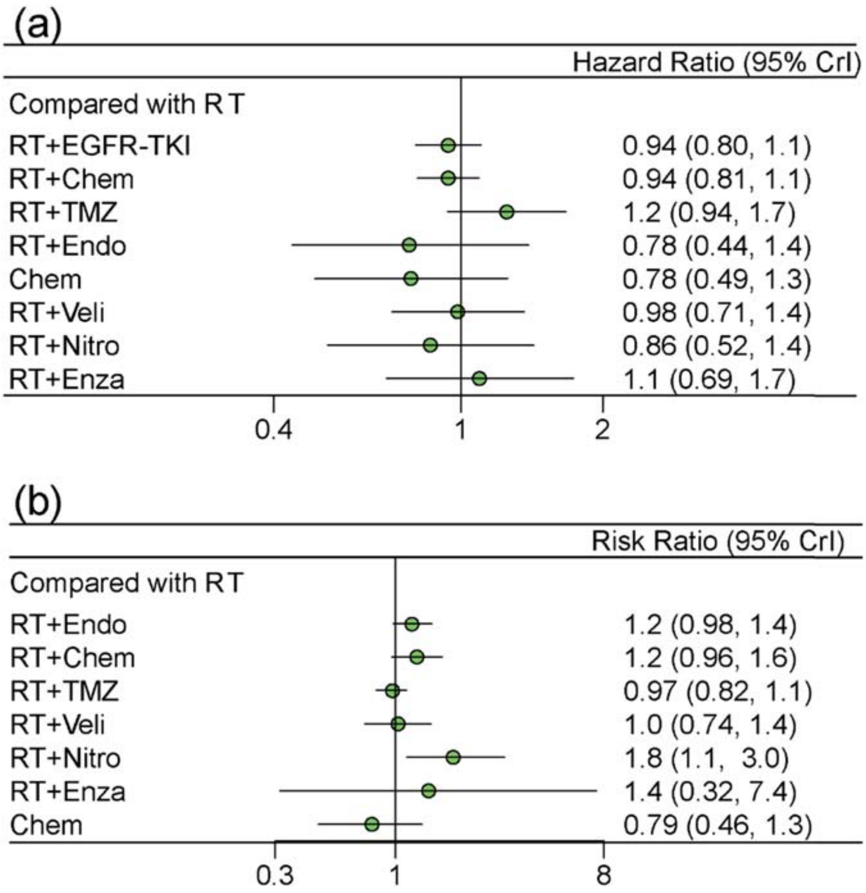

Figure S4: Forest plot for the sensitivity analysis of (a) overall survival and (b) objective response rate.

Table S1: The search strategy in detail

#1	Carcinoma, non-small-cell lung [Mesh]
non small cell lung cancer	NSCLC [title/abstract]
	Non small cell lung cancer [title/abstract]
	Carcinoma, non small cell lung [title/abstract]
	Carcinomas, non-small-cell lung [title/abstract]
	Lung carcinoma, non-small-cell [title/abstract]
	Lung carcinomas, non-small-cell [title/abstract]
	Non-small-cell lung carcinoma [title/abstract]
	Non small-cell lung carcinoma [title/abstract]
	Non-small-cell lung carcinoma [title/abstract]
	Non small cell lung carcinoma [title/abstract]
	Carcinoma, non-small cell lung [title/abstract]
	Non-small cell lung carcinoma [title/abstract]
	Non-small-cell lung cancer [title/abstract]
	Non-small cell lung cancer [title/abstract]
	Non small-cell lung cancer [title/abstract]
	Nonsmall cell lung cancer [title/abstract]
#2	Metastasis [title]
metastasis	Metastases [title]
	Metastatic [title]
#3	Brain [title]
brain	CNS [title]
	Central nervous system [title]
	Cerebral [title]
	Intracranial [title]
	Leptomeningeal [title]
#4	Randomized controlled trial [publication type]
RCT	Controlled clinical trial [publication type]
	Placebo [title/abstract]
	Group [title/abstract]
	Random [title/abstract]
	Randomized [title/abstract]
	Randomized [title/abstract]
	Randomly [title/abstract]
	Trial [title/abstract]
	Phase [title/abstract]

Table S2: Characteristics of included trials in terms of neurosurgery

Author/Year	Country	No. of patients (I/ No C)	Males%	Age †	Intervention arm	Control arm
Kim [1] 2015	Korea	11-Nov	81.8%/ 90.90%	65.6 (50–81)/ 61.9 (40–76)	Surgery+ Radiotherapy	Radiotherapy
Mandell 1986 [2]	US	35/69	63%/ 66%	52 (33–68)/ 58 (30/70)	Surgery+ Radiotherapy	Radiotherapy
Bougie [3] 2015	Canada	43/72	42%/ 49%	60 (39–84)/ 62 (42–86)	Surgery	SRS
Prabhu 2017 [4]	US	157/66	NA	58 (48–66)/ 59.5 (51–68)	Surgery+SRS	SRS
Yen 2021 [5]	China	23/213	56.5%/ 53.50%	60.0 (41–77)/ 62.7 (31–85)	Surgery+ Radiotherapy	Radiotherapy

Table S3: Global and local heterogeneity

Comparsion	OS	CNS-PFS	ORR
Global heterogeneity			
RT associated therapies	0%	0%	8%
ICIs	22%	33%	25%
Local heterogeneity			
Nivo+Ipil vs Chem	63.5%	NA	NA
Nivo vs Chem	0.0%	NA	NA
RT+Chem vs RT	9.3%		
RT+EGFR-TKI vs RT	18.1%	0.0%	NA
RT+Endo vs RT	0.0%	NA	0.0%
RT+TMZ vs RT	0.0%	NA	0.0%
RT+TMZ vs RT+EGFR-TKI	0.0%	NA	NA

References

[1] Kim SY, Hong CK, Kim TH, Hong JB, Park CH, Chang YS, et al. Efficacy of surgical treatment for brain metastasis in patients with non-small cell lung cancer. Yonsei Med J. 2015;56(1):103–11.

[2] Mandell L, Hilaris B, Sullivan M, Sundaresan N, Nori D, Kim JH, et al. The treatment of single brain metastasis from non-oat cell lung carcinoma. Surgery and radiation versus radiation therapy alone. Cancer. 1986;58(3):641–9.

[3] Bougie E, Masson-Côté L, Mathieu D. Comparison between surgical resection and stereotactic radiosurgery in patients with a single brain metastasis from non-small cell lung cancer. World Neurosurg. 2015;83(6):900–6.

[4] Prabhu RS, Press RH, Patel KR, Boselli DM, Symanowski JT, Lankford SP, et al. Single-fraction stereotactic radiosurgery (SRS) alone versus surgical resection and SRS for large brain metastases: a multi-institutional analysis. Int J Radiat Oncol Biol Phys. 2017;99(2):459–67.

[5] Yen CT, Wu WJ, Chen YT, Chang WC, Yang SH, Shen SY, et al. Surgical resection of brain metastases prolongs overall survival in non-small-cell lung cancer. Am J Cancer Res. 2021;11(12):6160–72.
